# Regulation of Rat Intrapulmonary Arterial Tone by Arachidonic Acid and Prostaglandin E2 during Hypoxia

**DOI:** 10.1371/journal.pone.0073839

**Published:** 2013-08-27

**Authors:** Gaoliang Yan, Qingjie Wang, Hui Shi, Yeshan Han, Genshan Ma, Chengchun Tang, Yuchun Gu

**Affiliations:** 1 Department of Cardiology, Zhongda Hospital of Southeast University Medical School, Nanjing, China; 2 Department of Anaesthesiology, Changzhou No. 2 People’s Hospital, Changzhou, China; 3 Institute of Molecular Medicine, Peking University, Beijing, China; University of Southampton, United Kingdom

## Abstract

**Aims:**

Arachidonic acid (AA) and its metabolites, prostaglandins (PG) are known to be involved in regulation of vascular homeostasis including vascular tone and vessel wall tension, but their potential role in Hypoxic pulmonary vasoconstriction (HPV) remains unclear. In this study, we examined the effects of AA and PGE2 on the hypoxic response in isolated rat intrapulmonary arteries (IPAs).

**Methods and Results:**

We carried out the investigation on IPAs by vessel tension measurement. Isotetrandrine (20 µM) significantly inhibited phase I, phase IIb and phase IIc of hypoxic vasoconstriction. Both indomethacin (100 µM) and NS398 attenuated KPSS-induced vessel contraction and phase I, phase IIb and phase IIc of HPV, implying that COX-2 plays a primary role in the hypoxic response of rat IPAs. PGE2 alone caused a significant vasoconstriction in isolated rat IPAs. This constriction is mediated by EP4. Blockage of EP4 by L-161982 (1 µM) significantly inhibited phase I, phase IIb and phase IIc of hypoxic vasoconstriction. However, AH6809 (3 µM), an antagonist of EP1, EP2, EP3 and DP1 receptors, exerted no effect on KPSS or hypoxia induced vessel contraction. Increase of cellular cAMP by forskolin could significantly reduce KPSS-induced vessel contraction and abolish phase I, phase II b and phase II c of HPV.

**Conclusion:**

Our results demonstrated a vasoconstrictive effect of PGE2 on rat IPAs and this effect is via activation of EP4. Furthermore, our results suggest that intracellular cAMP plays dual roles in regulation of vascular tone, depending on the spatial distribution of cAMP and its coupling with EP receptor and Ca^2+^ channels.

## Introduction

The normal pulmonary circulation is a low pressure and low resistance system with little or no resting vascular tone. Oxygen tension is a major mediator in determining pulmonary vascular tone. Unlike the systemic arterials which dilate in response to hypoxia, the pulmonary artery constricts when oxygen tension is lowered, a phenomenon known as hypoxic pulmonary vasoconstriction (HPV) [1]. For example, in human subjects as well in animals, exposure to hypoxic gas (10% O_2_) causes an increase in pulmonary arterial pressure with minimal change in the left atria pressure. HPV is restricted to the segments of the vasculature perfusing the poorly ventilated (or hypoxic) part of the lung, thereby maintaining an appropriate ventilation/perfusion ratio. HPV has been demonstrated in isolated resistance pulmonary artery rings [2] and even in isolated smooth muscle cells of the resistance pulmonary arteries (PASMC) [3]. HPV persists after lung denervation [4], in the absence of blood [5] and after endothelial denudation [6], suggesting that the core mechanism of HPV seem intrinsic to the PASMC, although it has been shown that HPV is partly mediated by the endothelium [7]–[9].

Although the mechanism responsible for HPV has still not been fully elucidated, an increase of [Ca^2+^]_i_ is a primary event in the contraction of PASMC. Ca^2+^ entry via voltage-gated Ca^2+^ channels (VGCC) [10] and voltage-independent Ca^2+^ channels [11], [12] have been shown to participate in HPV. On contrary, the role of Ca^2+^ release from the store via Ryanodine receptors in HPV remains controversial despite several previous reports suggesting that Ca^2+^ release may be essential to HPV [13]–[15].

Prostaglandins (PGs) are the products of arachidonic acid (AA) through reactions catalysed by phospholipase A2 (PLA2), cyclooxygenase(COX) and specific terminal PG synthases. A diverse family of PGs has been identified, including PGE2, PGF_2α_, PGD2, PGI2 and thromoboxane A2 [16]. AA and its metabolites are known to be important in regulation of local vascular tone, and their actions are mediated by a family of 8 G protein-coupled receptors designated EP 1–4 (for E-prostanoid receptor), FP, DP, IP, and TP. Altered prostanoid signalling has been implicated in chronic pulmonary diseases. For example, COX-2 expression has been shown to increase during hypoxia [17]. COX-2 null mice develop severe pulmonary hypertension with enhanced endothelial receptors. COX-2 deficient PASMCs gave a maladaptive response to hypoxia manifested by exaggerated contractility [18], which may be rescued by either PGI2 or PGE2. A number of prostanoid analogs [19], [20] and PG receptor antagonists [21] have been employed in the treatment or gone into clinical trial for a variety of vessel diseases including pulmonary hypertension. However, the role of various prostanoids and prostanoid receptors in HPV has not been fully delineated.

In the present study, we have evaluated the Ca^2+^ entry pathways in the hypoxic response of isolated rat intrapulmonary arteries (IPAs). The potential role of AA and its metabolite PGE2 in hypoxic vasoconstriction were explored using vessel tension measurement. Our results demonstrate that PGE2 alone exerts vasoconstriction in rat pulmonary artery via activation of EP4. EP4 is involved in hypoxic vasoconstriction.

## Materials and Methods

### Ethics statement

Animal experiments conformed to the Guide for the Care and Use of Laboratory Animals published by the US National Institutes of Health (DHWE publication No. 96-01, revised in 2002) and was approved by the Ethics Review Board for Animal Studies of Institute of Southeast University, Nanjing.

### Isolated rat intrapulmonary artery (IPAs)

Male Wistar rats (250–350 g) were anaesthetized with sodium pentobarbital (55 mg/kg, ip) and killed by cervical dislocation. Rat lungs were quickly removed to a bath containing cold physiological salt solution (PSS) for dissection. Small intrapulmonary arteries (150–350 µm) were isolated from surrounding tissue, cut as rings and mounted in a temperature controlled myograph system (DanisMyo Technology A/S model: 610 M). The bath solution (composition in mM: NaCl, 118; NaHCO_3_, 24; MgSO_4_, 1; NaH_2_PO_4_, 0.435; glucose, 5.56; CaCl_2_, 1.8; KCl, 4) was gassed with 95% air/5% CO_2_ (pH 7.4) at 37°C. Each ring was initially stretched to give an optimal pressure of 30 mmHg and the preparation was allowed to stabilize for 60 min. A surgical thread was pulled through the artery to obtain denuded arteries which showed diminished relaxation response to 1 µM acetylcholine.

### Hypoxic protocol

**Figure 1:**
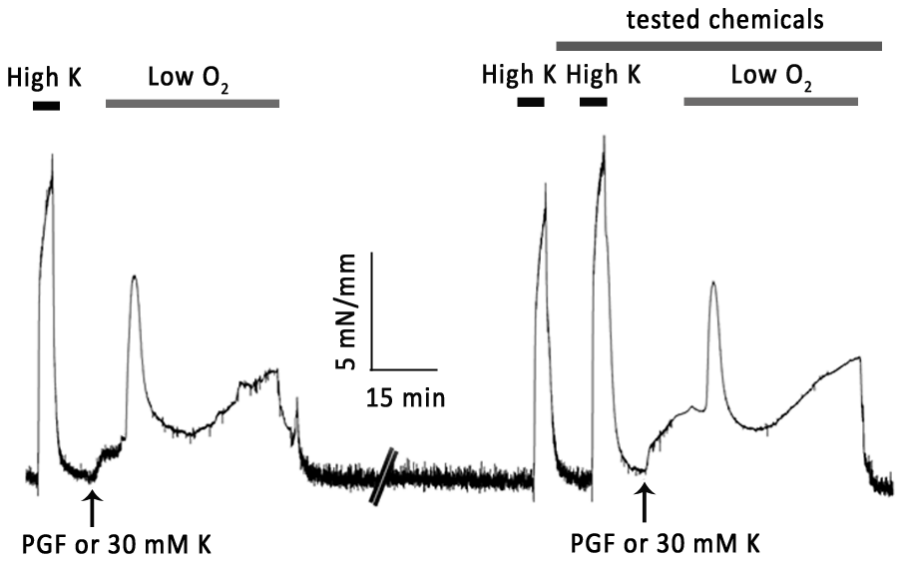
Repeatability of hypoxic responses in rat intrapulmonary arteries (IPAs). The IPAs were precontricted with 3–5 µM PGF_2α_, which equal to about 20% of high K^+^ response. The pretone was kept constant during experiments. The hypoxia was induced by bubbling myograph chamber with N_2_ balanced by 5% CO_2_ for 40 min, the O_2_ level was monitored by a dissolved oxygen meter, typically 1.5–2.5%. Then wash off by normal solution. After 1h recovery and two exposures to high K^+^, same hypoxia response was induced by following repeated protocol.

After a stabilization period of 60 min, IPAs were exposed to high KCl-PSS (KPSS) (composition in mM: NaCl, 42; NaHCO_3_, 24; MgSO_4_, 1; NaH_2_PO_4_, 0.435; glucose, 5.56; CaCl_2_, 1.8; KCl, 80) for 2min duration and this was repeated three times at 10min interval. 30 mM KPSS (in most experiments related to PG) or 5 µM of PGF_2α_ (in most experiments related to Ca^2+^ entry pathways) was added to the solution to achieve a resting tone equivalent to ∼15% of the high KPSS response. When the resting tone was stable, IPAs were exposed to hypoxia via bubbling the bath solution with a gas mixture of 95% N_2_ + 5% CO_2_ for 40 min, then washed with PSS and returned to normoxic condition. Oxygen level (∼19% in normoxia and ∼2% in hypoxia) was monitored using a dissolved oxygen meter in all experiments. Reproducible hypoxic responses could be obtained after 60–90min recovery time (figure1), so a standard 80 min recovery was observed between hypoxic challenges.

### Drugs

Chemicals were purchased from Biomol, except for verapamil (Sigma) and NS398 (Calbiochem). They were dissolved in ethanol, DMSO or distilled water as stock solution according to manufacturer instructions. Chemicals were diluted to the final concentration just before experiments. Ethanol and DMSO at same concentration were without effect.

### Statistical Analysis

**Figure 2:**
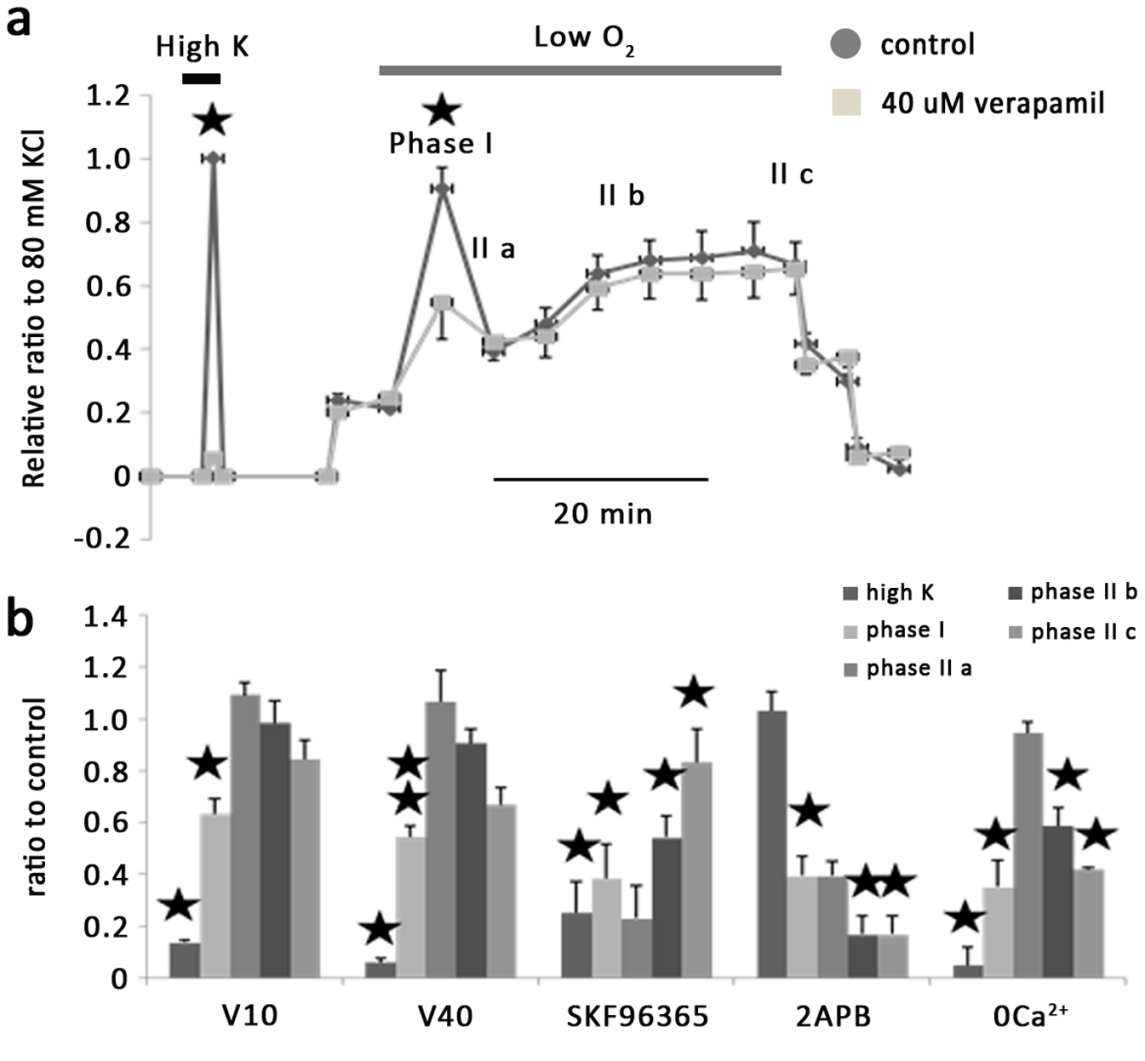
Effects of 40 µM verapamil on hypoxia induced vessel contraction in IPAs. a. Example figure of the effect of 40 µM verapamil on high K^+^ and hypoxia-induced smooth muscle constriction. HPV consisted of a transient constriction (phase I) superimposed on a sustained constriction (phase II), and phase II was divided into IIa, IIb and IIc. b. Mean data showing effect of 10 µM verapamil, 40 µM verapamil, 50 µM SKF96365 and 50 µM 2-APB on hypoxic induced vasoconstriction in IPAs in term of percentage change in compare with corresponding parts in control group. *, *P*<0.05, **, *P<*0.001. For 10 µM verapamil (n = 4), high K^+^ response and phase I hypoxic constriction reduced to 13.7±1% and 63.3±5.9% respectively. For 40 µM verapamil (n = 6), high k response and phase I hypoxic constriction reduced to 6.1±1.6% and 54.4±4.3% respectively. SKF96365 (n = 3): high K^+^, phase I, phase II b and c has been reduced to 25.3±11.7%, 38.6±13%, 54.2±8.3% and 83.3±12.3% respectively. 2APB (n = 3), which has no effect on high K, reduce phase I, phase II b and c to 39.5±7.5%, 16.9±7% and 16.9±7% and 0 calcium solution (n = 3)reduced high k, phase I, phase II b and c to 5±7%, 35.1±10.4%, 58.6±7% and 42.1±0.7% of controls. All results normalized using max vasoconstriction induced by 80 mM high k in control group.

IPA ring tension was normalized to the maximal tension induced by 80 mM KPSS in control group. Hypoxic vasoconstriction phase was divided as shown in figure 2a. Mean data shown in the bar graphs were expressed as a percentage change over the respective control. Results are shown as mean±SEM and compared using paired Student’s *t* test or Nonparametric Wilcoxon Signed Ranks test with a P value of less than 0.05 indicating statistical significance.

## Results

### 1. Effects of Ca^2+^ channel blockers on hypoxia-induced vasoconstriction in IPAs

In pilot experiments, we examined the reproducibility of the hypoxia-induced vasoconstrictive response of IPAs and found that consistently with a previous report [22] comparable responses could be obtained with a recovery period of 60–90 minutes between hypoxic episodes. Therefore, in later experiments a standard 80 min recovery period was allowed between hypoxic challenges. Hypoxia-induced vasoconstriction consisted of a transient constriction (phase I) superimposed on a sustained constriction (phase II), and phase II was divided into IIa (initial point of phase II), IIc (termination point of phase II) and IIb (average of time-points between IIa and IIc). Test chemicals were applied during the second hypoxic stimulation. Prior to the second hypoxic challenge, the basal tone was adjusted to the same level as that in the first hypoxic challenge by adding 30–40 mM KPSS or PGF2α (3 – 100 µM). PGF2α concentration needed to be raised in Ca^2+^ free solution (100 uM) and in the presence of Verapamil (3.5–4 uM) to achieve a pretone level [23]. To exclude the different effects due to the different the pretone methods, 30–40 mM KPSS and PGF2α (3 – 100 µM) were tested prior to hypoxia in one experiment, and consistent HPV was observed.

Verapamil was employed to determine the contribution of voltage-gated Ca^2+^ channel (VGCC) in hypoxia-induced vasoconstriction in IPAs. At a concentration of 10 µM (n = 5) or 40 µM (n = 6), verapamil (figure 2) almost abolished 80 mM KPSS-induced vasoconstriction (to 13.7±1% and 6.1±1.6% of controls, respectively), but only caused partial inhibition of the phase I hypoxic vasoconstriction (to 63.3±5.9% and 54.4±4.3% of controls, respectively). Neither concentrations of verapamil had significant effect on the amplitude of the phase II hypoxic constriction.

SKF96365 (50 µM), a non-specific Ca^2+^ blocker, significantly attenuated the contractile response to 80 mM KPSS to 25.3±11.7% of the control response. Hypoxia-induced vasoconstriction was also inhibited. Thus in the presence of SKF96365, phase I, phase II b and c of hypoxic vasoconstriction was decreased to 38.6±13%, 54.2±8.3% and 83.3±12.3% of the respective control value (n = 4). In contrast, 2APB (50 µM), an inhibitor of store-operated channels, exerted no effect on KPSS-induced contraction but inhibited phase I, phase IIb and phase II c of hypoxic vasoconstriction to 39.5±7.5%, 16.9±7% and 16.9±7% of controls (n = 4).

Our result also showed that Ca2+ free bath medium (n = 4) abolished KPSS and reduced hypoxia-induced vasoconstriction, suggesting that Ca2+ entry plays a key role in the contractile response of IPAs to hypoxia. In addition, the results above also indicate that hypoxic vasoconstriction is mediated by Ca^2+^ entry via both voltage dependent and voltage-independent channels.

### 2. Effects of blockage of AA generation on hypoxic response in IPAs

**Figure 3:**
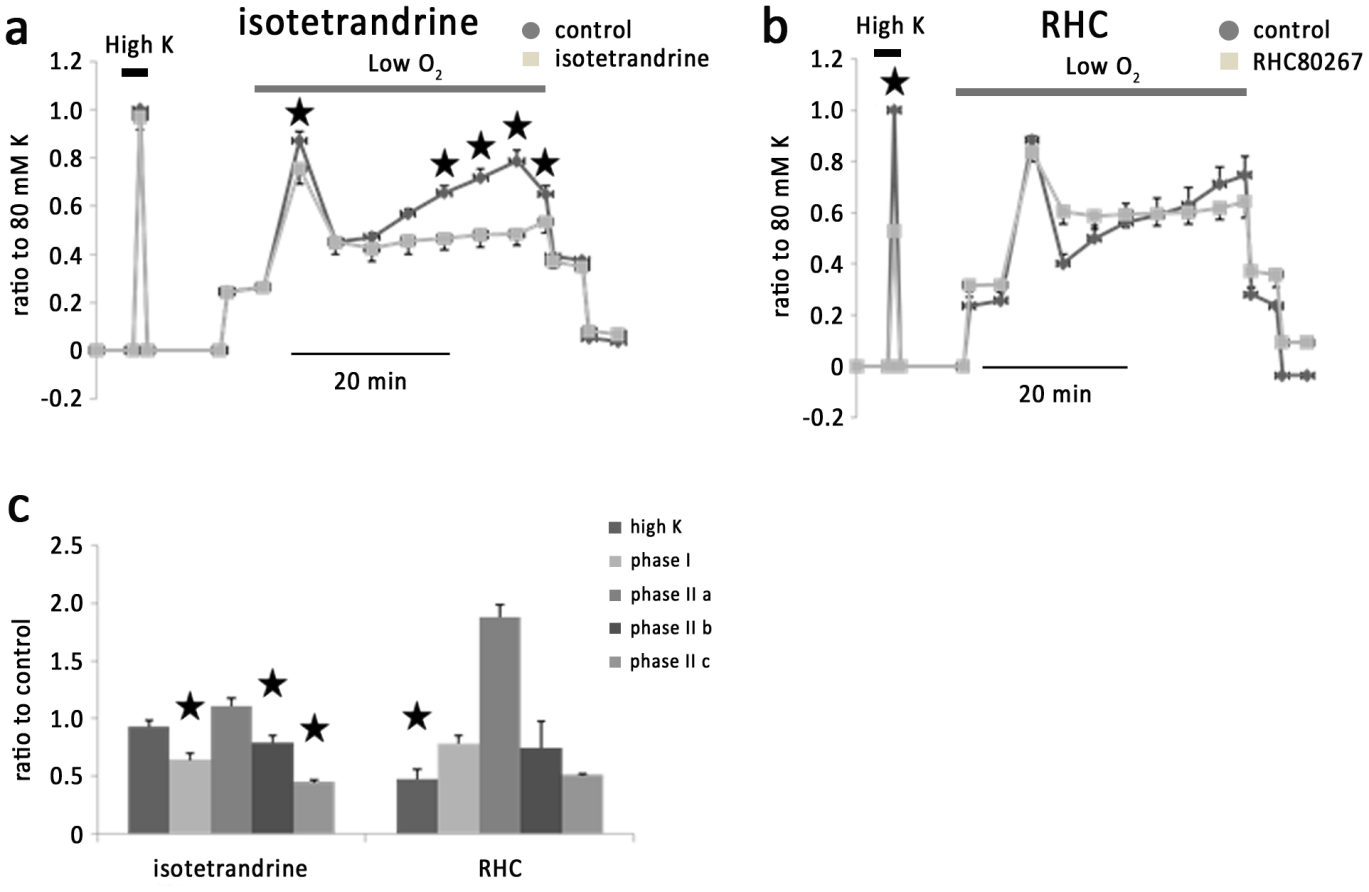
Effects of isotetrandrine and RHC80267 on vasoconstriction of IPAs. a. Isotetrandrine, an inhibitor of cPLA2, significantly inhibited phase I, phase II b and c of hypoxia induced vasoconstriction, but exerted no effect on KPSS induced vessel contraction. b. RHC80627, an inhibitor of DAG lipase, exerted significant inhibition on KPSS induced vasoconstriction but no effects on hypoxic response. c. Histogram showing the effects of isotetrandrine and RHC80267 on vasoconstriction of IPAs.

Previously it was shown that HPV was diminished in cPLA2-deficient mice [24], suggesting that AA and its metabolites may be important in the hypoxic response [25]. Isotetrandrine (20 µM), an antagonist of cPLA2, significantly inhibited phase I, phase II b and phase II c of hypoxic vasoconstriction to 64±5.7%, 78.9±6% and 44.6±1.9% of the controls respectively (n = 6) (figure 3a) without significant effect on KPSS-induced vasoconstriction. In contrast, RHC 80267 (50 µM), a DAG lipase inhibitor, inhibited KPSS-induced vasoconstriction by47.1±8.4% (n = 4) (figure 3b) without significant effect on hypoxic vasoconstriction. These results suggest that cPLA2 may be the primary pathway to generate AA in IPAs and endothelium is the primary targeting place by AA and its metabolites.

### 3. Effects of COX inhibitors on hypoxic vasoconstriction in IPAs

AA is converted to PGH2 by one of three isoforms of cyclooxygenase, namely COX-1, COX-2 and COX-3. COX-1, which is constitutively expressed has been associated with the immediate effect of AA [26] and the basal levels of prostanoid production. COX-2 is induced in response to inflammatory cytokines and mediators, resulting in increased and sustained prostanoid release. COX-3 is constitutively expressed in the specific tissues with the highest level seen in the brain and heart. PGH_2_ is converted to other prostanoids via distinct PG synthases, for example, PGE-synthase (PGES) for PGE_2_.

**Figure 4:**
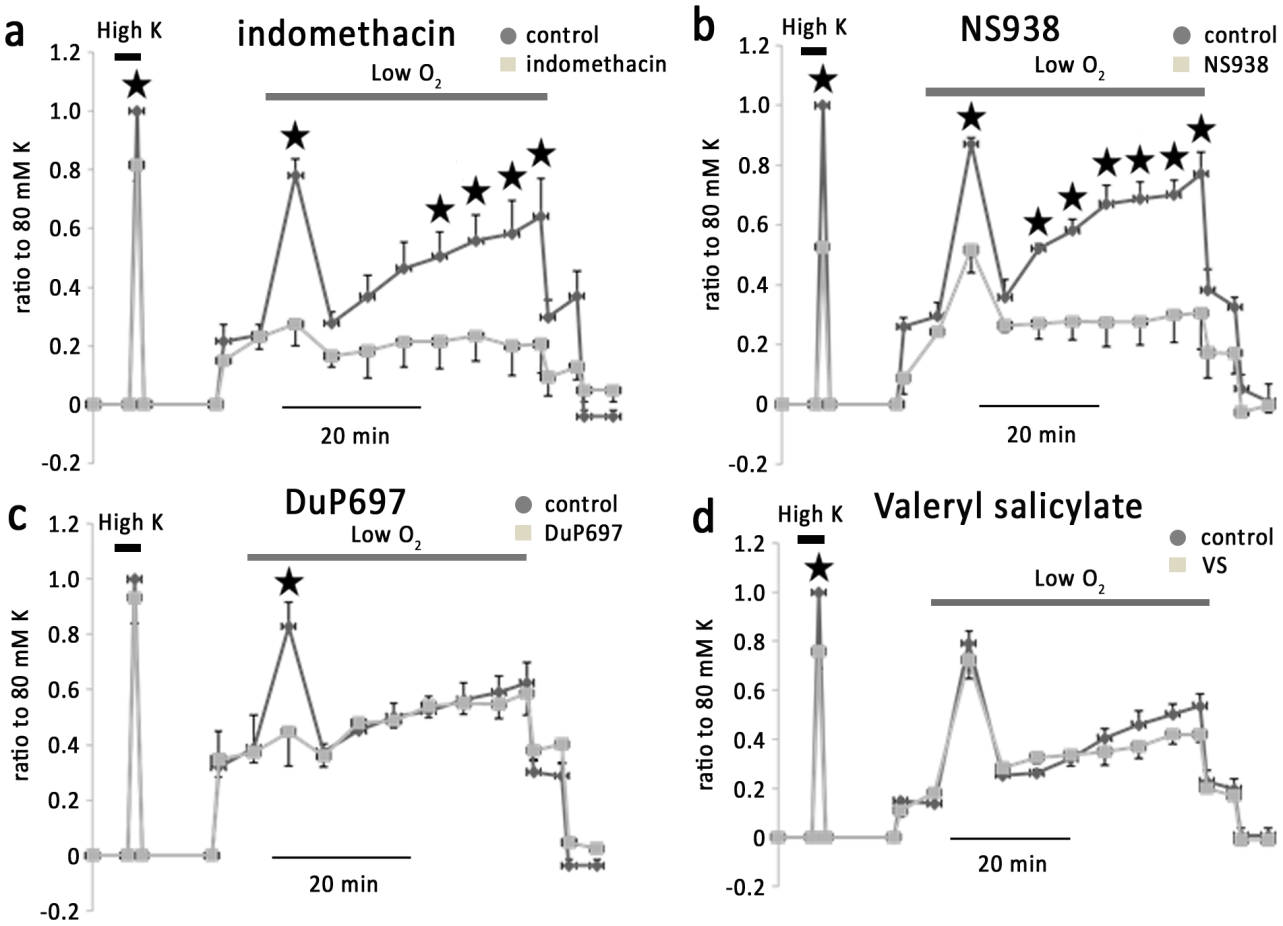
Effect of blockage of COX on hypoxic vasoconstriction in IPAs. a. Indomethacin, a nonspecific antagonist of COX, almost abolished hypoxic vasoconstriction in IPAs and exerted slight inhibition on KPSS induced vessel contraction. b. NS398, a specific antagonist of COX-2, abolished phase II of hypoxic vasoconstriction and significantly inhibited phase I of hypoxic vasoconstriction. c. DuP697, a highly selective and irreversible inhibitor of COX-2, abolished phase I of hypoxic vasoconstriction. d. Valeryl Salicylate, a highly selective antagonist of COX-1, attenuated KPSS induced vessel contraction.

Indomethacin (100 µM), a non-selective COX inhibitor, diminished the phase I phase II b and phase II c hypoxic vasoconstriction in IPAs by 90.8±9.3%, 97.7±9% and 108±10%, respectively (n = 5) (figure 4a). KPSS-induced vasoconstriction was only slightly inhibited by this concentration of indomethacin. NS398 (10 uM), a cell-permeable selective inhibitor of COX-2 [27], significantly attenuated phase I, phase II b and phase II c hypoxic vasoconstriction by 59.6±10%, 78.8±10% and 71±9% respectively (n = 5) (figure 4b), whereas valeryl salicylate (3 mM), a selective COX-1 inhibitor, was without significant effect on hypoxic vasoconstriction. DuP697 (25 µM), which is more potent on COX-2 than on COX-1, attenuated phase I hypoxic vasoconstriction by 83.2±10% (n = 5) (figure 4c) without significant effect on phase II hypoxic responses. NS398 and valeryl salicylate, at the above concentrations, inhibited KPSS-induced vasoconstriction by 46.2+3.2%, 23.4+6.9%, respectively. These results suggest that products via COX-2 mediate the hypoxic response of IPAs.

### 4. Effects of various EP receptor inhibitors on hypoxia-induced vasoconstriction in IPAs

At the present, a family of 8 GPCRs have been identified to be responsible for the diverse effects of different prostanoids. They are designated EP 1–4 (for PGE2), FP, DP, IP, and TP, respectively. To investigate the prostanoid receptor subtypes that are involved in the hypoxia-induced vasoconstriction of IPAs, we utilised different prostanoid receptor antagonists.

**Figure 5:**
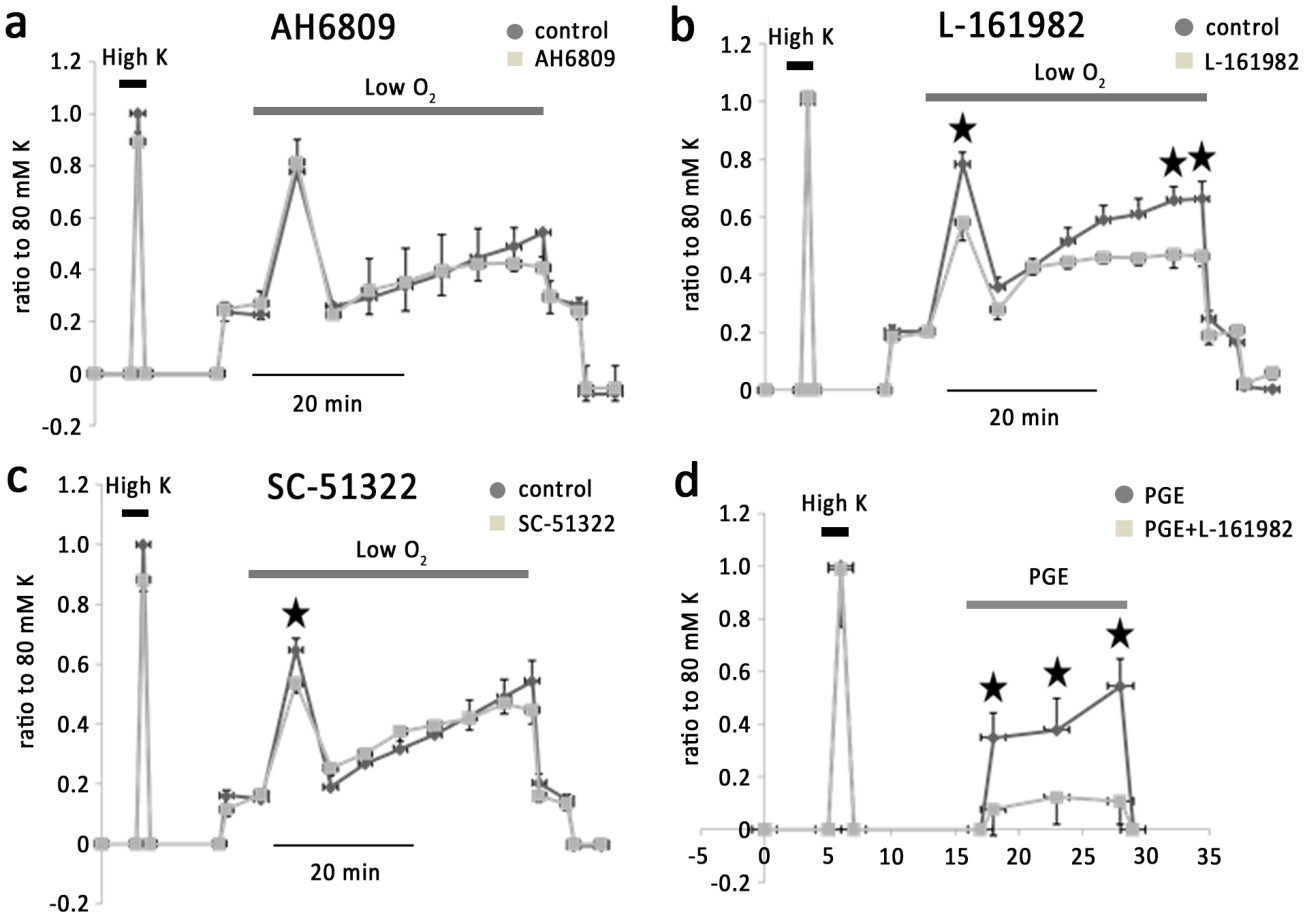
Effects of various EP receptor inhibitors on hypoxic vasoconstriction in IPAs. a. AH6809, a general blocker of EP1, EP2, EP3 and DP1 receptors, exerted no effect on hypoxic vasoconstriction. b. L-161982, an antagonist of EP4, significantly inhibited phase I and phase II b and c. c. SC-51322, an antagonist of EP1 receptor, inhibited phase I of hypoxic vasoconstriction. d. L-161982 inhibited PGE induced vasoconstriction but not KPSS induced vessel contraction.

**Figure 6:**
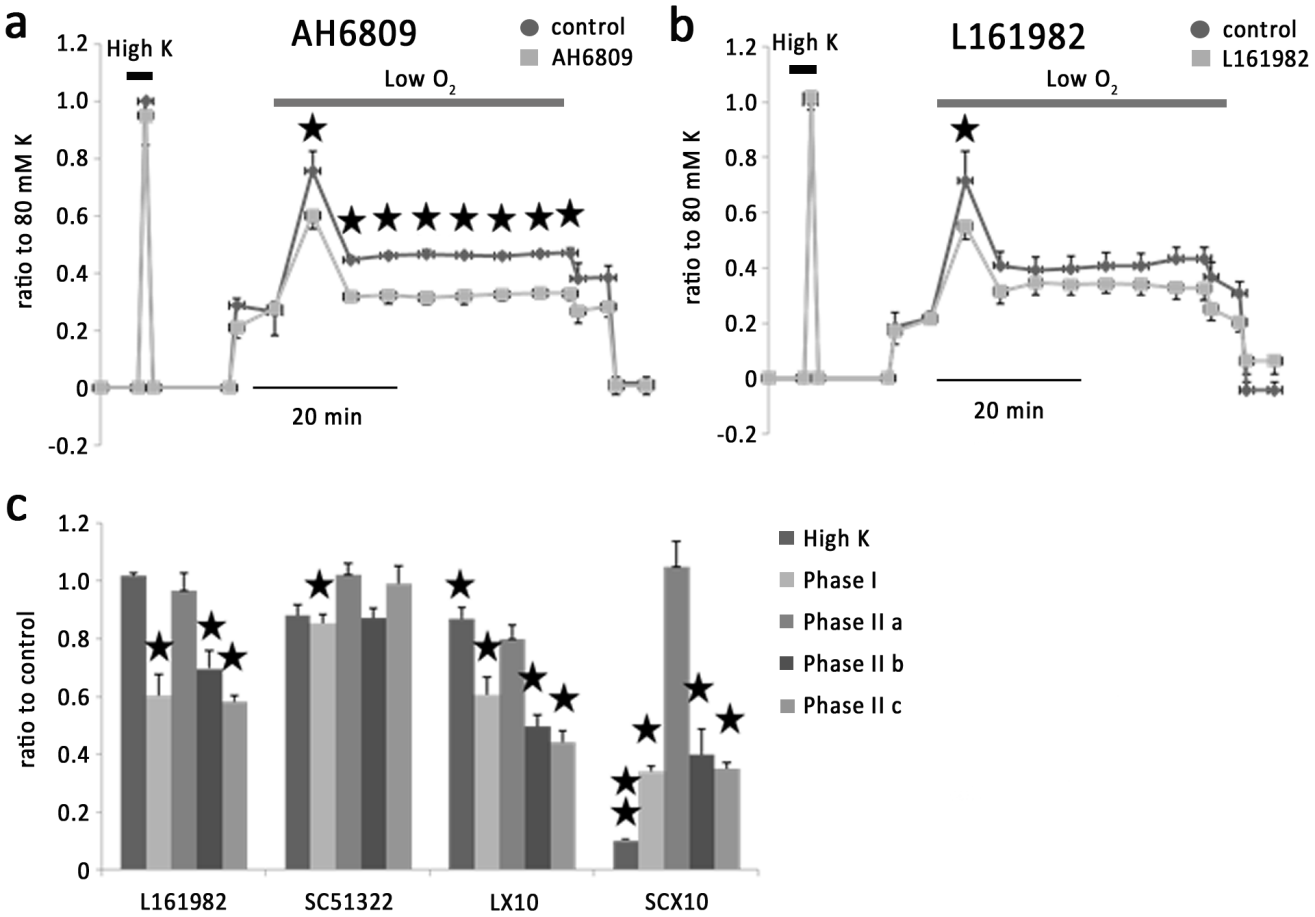
Effect of AH6809 and L161982 on hypoxic pulmonary vasoconstriction in denuded IPAs. a. Transient hypoxic vasoconstriction was suppressed by AH6809, and L-161982. b. Histogram showing the effects of different dosages of chemicals above on hypoxic pulmonary vasoconstriction.

PGE alone caused vasoconstriction. SC-51322 (1 µM), a potent antagonist of EP1–4 receptors, inhibited phase I hypoxic vasoconstriction by 14.4±3% (n = 6) (figure 5c). At a concentration of 10 µM SC-51322 caused a strong inhibition of KPSS and hypoxia-induced vessel contraction (figure 6c). AH6809 (3 µM), which blocks EP1-3 but not EP4, was without effect on KPSS or hypoxia-induced vessel contraction (n = 6) (figure 5a). These observations suggest possible involvement of EP4 in the hypoxic response of IPAs. This is confirmed by using L-161982, a selective antagonist of EP4 receptor. L-161982 (1 µM) inhibited phase I, phase II b and phase II c hypoxic vasoconstriction by 39.5±7.3%, 30.3±6.2% and 41.7±2.1% respectively (n = 6) (figure 5b). At this concentration, L-161982 inhibited PGE2-induced vasoconstriction by an average 69.1±5.6% (n = 5) without affecting KPSS-induced contraction (figure 5d). Denuded IPAs were used to eliminate PGs generated by endothelium. In denuded IPAs, the effect of L161982 was similar to that on intact IPAs (figure 6b). In contrast, AH6809 which had no effect on intact IPAs, caused a strong inhibition of the phase I, phase II a, phase II b and phase II c hypoxic vasoconstriction by 36.4 ±3.6%, 78.4±10%, 79.7±7.1% and 76.6±9.3% respectively (n = 6) (figure 6a). These results suggest that PGE2 regulate vessel tone during hypoxia via interacting with EP4 receptor.

### 5. Role of cAMP in hypoxic response in IPAs

**Figure 7:**
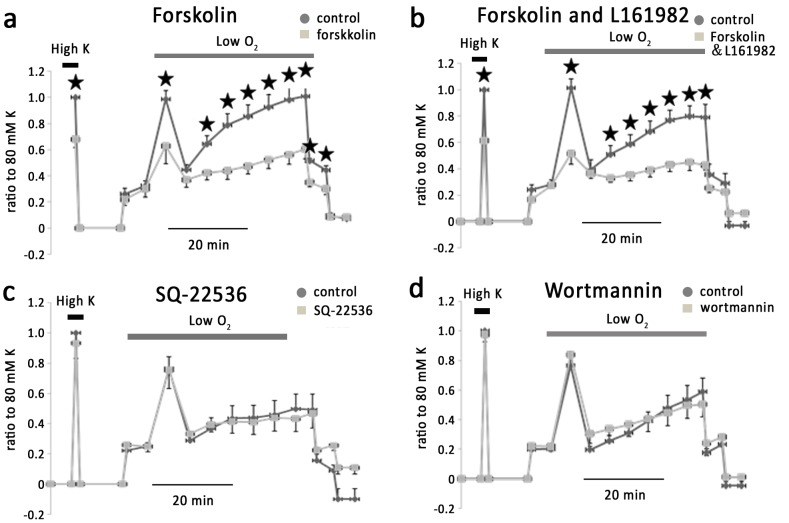
Hypoxic vasoconstriction in IPAs was independent on PIK3 but attenuated by cellular cAMP. a. Forskolin significantly inhibited the phase I, phase II b and c of vasoconstriction respectively. b. Forskolin with L161982 further attenuated hypoxic vasoconstriction in IPAs. c. SQ-22536, an inhibitor of cAMP production induced by PGE1, exerted no effect on either KPSS or hypoxia induced vessel contraction. d. Wortmannin, an antagonist of PI_3_K, exerted no effect on vessel contraction.

Activation of EP4 has been shown to increase intracellular cAMP via stimulating adenylatecyclase. We therefore examined the effect of Forskolin (10 µM), an activator of adenylate cyclase and found that it significantly reduced KPSS-induced vessel contraction by 38.6±4.9%, and phase I, phase II b and phase II c hypoxic vasoconstriction by 60.5±10%, 54.2±10% and 58.5±5.1% respectively (n = 5) (figure 7a). The inhibitory effect of forskolin was not prevented by L-161982 (figure 7b) (n = 6). However, SQ-22536 (85 µM), a cell-permeable adenylate cyclase inhibitor, had no effect on KPSS or hypoxia-induced vessel contraction (n = 6) (figure 7c), arguing against the involvement of cAMP pathway in these responses

Previous studies have shown bradykinin stimulated PGE2 release in cultured human pulmonary artery smooth muscle cells, which is inhibited by PI_3_K blocker [28]. However, in our preparation PI_3_K blocker wormannin (50 nM) failed to alter the basal vascular tone or hypoxic vasoconstriction (figure 7d).

## Discussion

In this study, isolated rat IPAs exhibited biphasic contractile responses to prolonged hypoxia (40 min), consisting of a transient phase I constriction superimposed on a sustained phase II constriction, which has been shown to be endothelium-dependent. Using different Ca^2+^ channel blockers, we show that Ca^2+^ entry via voltage-gated and voltage-independent channels were both important in hypoxic vasoconstriction. Inhibitors of AA generation via cPLA2 or prostanoid synthesis via COX-2 significantly attenuated hypoxia-induced vessel contraction. PGE alone could initiate vasoconstriction. Antagonists that had efficacy on EP4 receptor could partly inhibit the hypoxic response. These observations suggest that AA metabolites are important players in regulation of hypoxic vasoconstriction and that EP4 appear to be involved.

In order to examine the contributions of Ca^2+^ entry pathways in the hypoxic vasoconstriction of IPAs, we applied varying concentrations of PGF_2α_ to adjust the baseline vessel tension to the same level. Previous studies have shown that 0.1–1 µM PGF_2α_ resulted in a transient followed by a plateau rise in [Ca^2+^]_i_ [29] via activation of FP receptor. This [Ca^2+^]_i_ elevation was composed of Ca^2+^ release from store and Ca^2+^ entry via store operated Ca^2+^ entry (SOC). Higher concentrations (10 µM) of PGF_2α_ led to Ca^2+^ entry via L-type VGCCs and receptor operated Ca2+ channels as a result of TP receptor activation. Noticeably, the presence of varying concentrations of PGF2α may have influence on the hypoxic responses and thus may bias interpretation of our data, when experiments were performed to investigate effects of EPs. We therefore have performed experiments by using varying concentrations of KPSS (30–40 mM KCl) to adjust the baseline tone. In addition, vessel contraction elicited by 80 mM KPSS prior to and after application of chemicals was compared. The significant difference in KPSS response with or without tested chemicals indicates the modulation of VGCCs by applied chemicals.

Consistent with previous reports [23], our results also show incubation in Ca^2+^-free bath medium abolished hypoxic vasoconstriction in the rat IPAs, confirming the key role of Ca^2+^ entry in the hypoxic response. 2-APB and SKF96365 were employed to identify the role of Ca^2+^ entry via voltage-independent channels in hypoxic vasoconstriction. They both exert an inhibitory effect on voltage independent Ca^2+^ influx. SKF-96365 not only inhibits voltage-independent Ca^2+^ entry e.g. TRP channel, but also attenuates voltage dependent Ca^2+^ entry, which is interpreted by its effect on decrease of 80 KPSS induced vasoconstriction, as vearapmil almost abolishes the 80 mM KPSS induced vasoconstriction. 2 APB as a membrane permeable SOCE inhibitor exerts no effect on KPSS induced contraction but attenuates hypoxic phase I and phase II constriction. In conclusion, SKF96365 works on both voltage dependent and voltage independent Ca2+ entry. 2APB in dosage (50 µM) has more specificity on SOCE. In addition to VGCC, voltage independent Ca2+ entry contributes to HPV. It is noticeable that SKF96365 exerted a similar inhibition effect in the phase I of HPV as 2-APB performed, but had less inhibition in the phase II of HPV than 2-APB did. It is well documented that phase II of HPV is endothelium dependent and voltage-independent Ca2+ channel is predominant in endothelium, suggesting that SKF96365 in compare with 2APB is less favour on voltage-independent Ca2+entry.

A variety of lipids including AA and its metabolites have been demonstrated to activate and/or modulate diverse channels including K channels [30], VGCCs [10], [31] and TRP channels [32], [33]. In our previous study, we have shown exogenous AA could attenuate hypoxia-induced Ca^2+^ elevation in cultured human pulmonary artery smooth muscle cells via inhibition on voltage-independent Ca^2+^ entry [34]. Here we show that in the rat IPAs, hypoxic vasoconstriction was inhibited by a blocker of cPLA2 which is involved in the production of AA. This observation is consistent with the observation on cPLA2 knockout mice which had diminished hypoxic pulmonary vasoconstrictitive responses [24] and suggest that endogenous AA is a mediator promoting hypoxic vasoconstriction in the rat pulmonary vessels. In addition, we found that DAG lipase inhibitors had no effect on the hypoxic response. Therefore, it appears that in the rat IPAs, AA largely derives from cPLA2 pathway.

Two major isoforms of COX have been identified in rat pulmonary artery, namely COX-1 and COX-2. COX1 is constitutively expressed and is associated with the basal level of prostanoid. Its product PGH2 is converted to PGE2 by cytosolic PGEs (cPGEs). COX-2 is induced in response to inflammatory cytokines and mediators, resulting in increased and sustained prostanoid release. PGH2 produced by COX-2 is converted to PGE2 via microsomal PGEs (mPGEs). We found that the non-selective COX inhibitor indomethacin significantly inhibited hypoxic vasoconstriction in rat IPAs and this effect was mimicked by the COX-2 selective inhibitor NS398. However, DuP697 only inhibit phase I responses, suggesting that COX-2-mPGEs-PGE2 pathway plays a primary role in regulating rat pulmonary arterial tone during hypoxia at least in phase I. The difference derived from the two cox-2 inhibitors is likely due to NS398 binding within the cyclooxygenase channel of COX-2 in a similar fashion to indomethacin, but in a different position for other COX-2-selective inhibitors [35]. In addition, NS398 is the specific blocker for COX-2 but DuP697 has effects on both COX2 and COX1. COX-1 and COX-2 are of similar molecular weight and have 65% amino acid sequence homology and near-identical catalytic sites. The selective inhibition is the substitution of isoleucine at position 523 in COX-1 with valine in COX-2. Drug molecules, such as DuP-697, bind to the alternative site to be considered in selective COX1 andCOX-2. Valeyl Salicylate which is a specific blocker of COX1 had little effect on HPV. It may suggested COX2 plays a key role in regulation of HPV. With respect to the different COX selectivity between NS398 and DuP697, different inhibition effects derived from them on the phase II of HPV implicated the expression COX2 may be higher in endothelium. The messengers released from endothelium regulate the signaling pathways of smooth muscle cells and profoundly contribute to HPV.

We further explored the prostanoid receptor subtypes that might be involved in hypoxic vasoconstriction in rat IPAs. We found that prostanoid receptor antagonists with efficacy on EP4 inhibited hypoxic vasoconstriction, whereas inhibitors of EP1-3 exerted no effect. AH6809 inhibited phase II in denuded IPAs, and it may be contributed by the lower pretone in the presence of AH6809. These results implicated that endogenous prostanoids, presumably PGE2 regulate vessel tone during hypoxia via interacting with EP4 receptor.

Activation of EP4 is reported to be coupled with an increase of cellular cAMP via Gs [26]. We therefore examined the possible role of cAMP in hypoxic vasoconstriction in IPAs. Surprisingly, forskolin, an activator of adenylyl cyclase caused significant attenuation of KPSS and hypoxia-induced vasoconstriction in rat IPAs, suggesting that a global cAMP elevation induced by forskolin, affected both voltage-dependent and voltage-independent Ca^2+^ channel [36]. In summary, we found that in the isolated rat IPAs, prostanoid signal pathway is an important mechanism in control of vessel tone during hypoxia. Upon hypoxia, AA produced via cPLA2 was broken down to PGE2 via COX-2 and mPGE pathway, and that PGE2 interact with EP4 leading to consequently vasoconstriction. ONO-AE-248 (EP3 agonist) induces vessel contraction [38] in human pulmonary artery, but EP3 antagonist exerts no effect on isolated rat IPAs, suggesting the different mechanisms underlining the vasoconstriction by PGE in different species [37]. The contracted human pulmonary arteries fail to dilate when they are challenged with PGE2 [39]. In addition, relaxation in human pulmonary vein by prostnoids involves DP, IP receptors and EP receptors. These observations signify the profound and complex role of prostanoid signaling in control of pulmonary vessel tone in different situations and species.
